# Epidemiologic study of clinically amyopathic dermatomyositis and anti-melanoma differentiation-associated gene 5 antibodies in central Japan

**DOI:** 10.1186/ar3547

**Published:** 2011-12-22

**Authors:** Yoshinao Muro, Kazumitsu Sugiura, Kei Hoshino, Masashi Akiyama, Koji Tamakoshi

**Affiliations:** 1 Division of Connective Tissue Disease and Autoimmunity, Department of Dermatology, Nagoya University Graduate School of Medicine, 65 Tsurumai-cho, Showa-ku, Nagoya 466-8550, Japan; 2 Department of Dermatology, Nagoya Ekisaikai Hospital, 4-66 Shonen-cho, Nakagawa-ku, Nagoya 454-8502, Japan; 3 Department of Nursing, Nagoya University School of Health Sciences, 1-1-20 Daiko-Minami, Higashi-ku, Nagoya 461-8673, Japan

## Abstract

**Introduction:**

Several reports have found the onset or activity of inflammatory myopathies to show spatial clustering and seasonal association. We recently detected autoantibodies against melanoma differentiation-associated gene 5 (MDA-5) in more than 20% of patients with dermatomyositis. Anti-MDA-5 antibodies were associated with the presence of rapidly progressive interstitial lung disease in clinically amyopathic dermatomyositis (CADM). The present study aims to assess the growing prevalence of CADM and the geographical incidence of anti-MDA-5-positive patients.

**Methods:**

We reviewed medical charts and examined the presence of anti-MDA-5 antibodies in 95 patients, including 36 CADM patients. Sera were obtained from 1994 through 2011. Statistical analyses were performed to assess whether CADM development and the presence of anti-MDA-5 antibodies were associated with various parameters, including age at disease onset, season of onset, annual positivity, and population of resident city.

**Results:**

Tertiles based on the year when the sera were collected showed increasing tendencies of CADM and anti-MDA-5-positive patients among all of the dermatomyositis patients. From 1994 to 2010, the relative prevalence of CADM and anti-MDA-5 antibody-positive patients significantly increased. Interestingly, the presence of anti-MDA-5 antibodies in 26 patients was inversely associated with the population of their city of residence.

**Conclusions:**

This is the first study to examine the distribution of anti-MDA-5-positive dermatomyositis phenotypes in Japan. Regional differences in the incidences of these phenotypes would suggest that environmental factors contribute to the production of antibodies against MDA-5, which triggers innate antiviral responses.

## Introduction

Idiopathic inflammatory myopathies are a heterogeneous group of autoimmune disorders that target the skeletal muscle and skin. Disease-related death is generally associated with malignancy and interstitial lung disease. The most frequent forms, polymyositis and dermatomyositis (DM), are thought to result from environmental exposure that leads to immune activation in genetically susceptible individuals. Several reports have found the onset or activity of inflammatory myopathies to show spatial clustering and seasonal association [1-5].

A subgroup of DM patients who have typical skin manifestations of DM but little evidence of myositis has been recognized as clinically amyopathic dermatomyositis (CADM) [6]. Although it is still undetermined whether CADM is a distinct clinical entity or just an early phase of classic DM, rapidly progressive interstitial lung disease (ILD) can occur in CADM patients, especially in East Asia [7]. This patient subset with CADM and rapidly progressive ILD has been shown to have specific autoantibodies, originally called anti-CADM-140 antibodies [8]. The target autoantigen is melanoma differentiation-associated gene 5 (MDA-5) [9-11], which plays important roles in the innate immune system during RNA virus infections [12].

To better understand this subset of patients, it is important to examine the epidemiologic characteristics of CADM patients with anti-MDA-5 antibodies, whose outcome is often fatal. According to our clinical experiences, we have recently noticed that the prevalence of CADM patients with anti-MDA-5 antibodies seems to be growing, particularly in rural areas. We therefore examined the epidemiologic features of CADM and anti-MDA-5 antibodies in a single cohort of DM patients.

## Materials and methods

### Patients

We reviewed medical charts and examined the presence of anti-MDA-5 antibodies in 95 Japanese patients (one of them a half-Japanese, half-Filipino boy) with DM, including 36 patients with CADM, 15 patients with cancer-associated DM and 44 patients with classical DM, who were seen by or consulted the Department of Dermatology at Nagoya University Graduate School of Medicine from 1994 to 2011. These patients were diagnosed with DM or CADM based on the criteria of Bohan *et al. *[13] or Sontheimer [6], respectively. In general, CADM presents as typical skin lesions and amyopathy or hypomyopathy that lasts for more than 6 months. The CADM group included patients who developed fatal ILD within the first 6 months after disease onset. Since juvenile DM with rapidly progressive ILD and/or anti-MDA-5 antibodies has been reported in Japan [7,11,14], patients who manifested the disease at < 18 years of age were also included. Patients who were originally seen at other hospitals far outside our area and who then transferred to our hospital were excluded from the present study. Serum samples were obtained from all of the patients between 1 October 1994, the date when we began to build a serum bank of autoimmune rheumatic disease patients, and 30 June 2011. The population data on city of residence in 2010 were obtained from web data published by public offices in 25 cities, eight counties and one village.

The present study was approved by the Ethics Committee of Nagoya University Graduate School of Medicine. This study meets and is in compliance with all ethical standards in medicine. Informed consent including that for publication of the study was obtained from all patients according to the Declaration of Helsinki.

### Immunoprecipitation

Anti-MDA-5 antibodies were screened by an immunoprecipitation assay using biotinylated recombinant MDA-5 produced from full-length MDA-5 cDNA using the TnT^® ^T7 Quick Coupled Transcription/Translation System (Promega, Madison, WI, USA) and the Transcend^™ ^Colorimetric Non-Radioactive Translation Detection System (Promega), according to our published protocol [11]. This method was confirmed to produce consistent results based on a standard immunoprecipitation assay using ^35^S-methionine-labeled cell extracts [11]. Serum samples from 82 patients were already characterized in our previous report [11]. All serum samples were stored at -70°C until the experiments.

### Statistical analysis

The subjects were divided into tertiles based on year the sera were collected, age at collection, age at onset, or population of the city of residence, separately, to examine the associations between each of these factors and the development of CADM and the presence of anti-MDA-5 antibodies. The differences and linear trends across the tertiles were assessed using the chi-square test and the Cochran-Armitage trend test, respectively. SPSS version 17.0 for Windows (SPSS Japan Inc., Tokyo, Japan) was used to perform the statistical analysis. *P *< 0.05 was considered significant.

## Results

### Patient population

Between 1 October 1994 and 30 June 2011, sera from 95 patients with DM were collected. During 1994 sera were drawn from 24 patients, two-thirds of whom had been diagnosed with DM and treated by our department. The mean age at onset was 46.9 years (range: 1 to 80 years) and that at the time of sera collection was 50.2 years (range: 3 to 84 years). There were 67 (70.5%) female patients. Ten patients developed the disease under 18 years of age.

A review of the medical records indicated that 36 patients (28/36, 77.8% female; 5/36, 13.9% juvenile) had CADM. For these 36 patients, the mean age at onset was 44.9 years (range: 1 to 73 years) and that at the time of sera collection was 48.2 years (range: 3 to 84 years). Based on the immunoprecipitation assays, 26 patients (21/26, 80.8% female; 1/26, 3.8% juvenile) had anti-MDA-5 antibodies. For these 26 patients, the mean age at onset was 46.8 years (range: 11 to 66 years) and that at the time of sera collection was 48.2 years (range: 11 to 71 years). Twenty-five patients with anti-MDA-5 antibodies were diagnosed as CADM, and the remaining patient met the criteria for classical DM. All but one of our patients with anti-MDA-5 antibodies had ILD.

To grasp the overall trend, tertile analysis was conducted based on the number of cases for all patients with DM as well as for patients with CADM and those with anti-MDA-5 antibodies (Table 1). The mean ages at onset and at the time of sera collection did not significantly differ among the tertiles (data not shown), but the proportions of CADM and anti-MDA-5-positive patients significantly increased from the first to the third periods of the study.

**Table 1:** Patient characteristics based on the presence of CADM or anti-MDA-5 antibodies

			CADM			α-MDA-5-positive		
			
Years of sera collection	Total number of DM patients (M:F)	Mean age at onset (range)	Number (%) of patients (M:F)	*P *value*	Mean age at onset (range)	Number (%) of patients (M:F)	*P *value**	Mean age at onset (range)
T1 (1994 to 1995)	32 (12:20)	47.5 (4 to 80)	6 (18.8%) (2:4)	*P *for difference = 0.012	45.5 (4 to 73)	2 (6.3%) (1:1%)	*P *for difference = 0.003	53 (43 to 63)
T2 (1996 to 2003)	30 (6:24)	50.1 (15 to 79)	12 (40.0%) (1:11)	*P *for trend = 0.003	50.7 (20 to 73)	10 (33.3%) (0:10)	*P *for trend = 0.001	48.9 (20 to 66)
T3 (2004 to 2011)	33 (10:23)	43.6 (1 to 73)	18 (54.5%) (5:13)		40.8 (1 to 69)	14 (42.4%) (4:10)		44.4 (11 to 58)

CADM, clinically amyopathic dermatomyositis; DM, dermatomyositis; M:F, male:female; MDA-5, melanoma differentiation-associated gene 5. *Prevalence of CADM in total DM. **Prevalence of anti-MDA-5 in total DM.

### Annual prevalence of CADM and anti-MDA-5 antibodies

**Figure 1:**
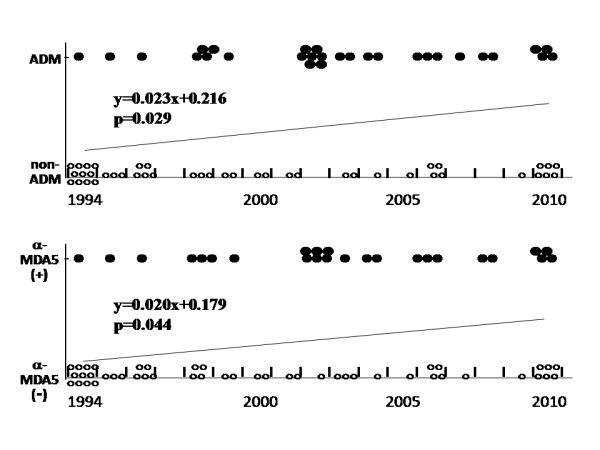
**Annual prevalence of patients with clinically amyopathic dermatomyositis or anti-melanoma differentiation-associated gene 5 antibodies**. The regression equation is shown, in which the year of disease onset is defined as 1994 = 1, 1995 = 2,..., 2010 = 17 on the *x *axis and the presence or absence of clinically amyopathic dermatomyositis (CADM) or anti-melanoma differentiation-associated gene 5 (anti-MDA-5) antibodies is defined as 1 and 0, respectively, on the *y *axis (*P *for linear trend).

Since many of the patients whose sera were drawn in 1994 had been treated at our hospital, the above tertile analysis was partially biased. Before the sampling in 1994 there may have been some fatal cases of rapidly progressive ILD. Moreover, some CADM patients stopped seeing their doctors due to minor illness. In light of these possibilities, we analyzed only the 72 patients who manifested the disease after 1994, in order to investigate the growing trend of CADM and anti-MDA-5-positive patients (Figure 1). The relative prevalence of both CADM and anti-MDA-5-positive patients among all DM patients was found to have significantly increased (*P *= 0.029 and *P *= 0.044, respectively).

### Geographical incidence of dermatomyositis patients with anti-MDA-5 antibodies

**Figure 2:**
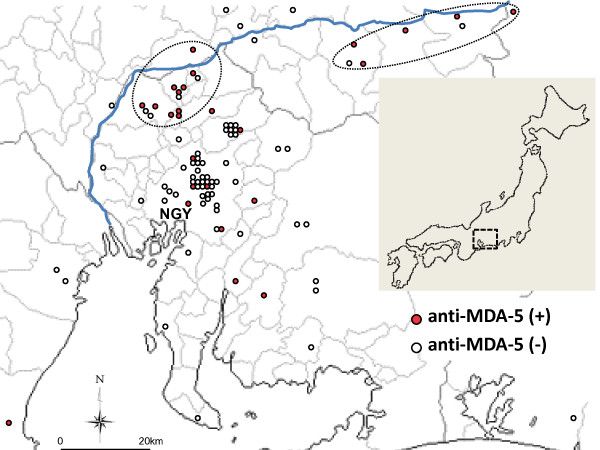
**Geographic distribution of patients with dermatomyositis**. A residential area of 95 patients was plotted. NGY, Nagoya city. Red and white circles show patients with and without anti-melanoma differentiation-associated gene 5 (anti-MDA-5) antibodies, respectively. Blue line shows the Kiso River, which is the biggest in the area.

Our university hospital is in Nagoya (population 2.2 million), the biggest city in central Japan. To clarify the regional differences in a subgroup of patients, we compared the prevalence of CADM and anti-MDA-5-positive patients by tertiles based on the population of the patient's city of residence (Table 2), and we plotted the anti-MDA-5-positive patients on a map (Figure 2). Interestingly, CADM patients were less prevalent in urban areas, but this association was only marginally significant, whereas there were significantly more anti-MDA-5-positive patients in rural areas than in urban ones. Areas northeast and far northwest of Nagoya contained particularly high numbers of patients with anti-MDA-5 antibodies: 10 patients in the northeast, and five patients in the northwest (Figure 2, circular dotted area). These areas had nine and six CADM patients in the northwest and northeast, respectively. All 15 patients with anti-MDA-5 antibodies were natives of the area; five and four of these 15 patients had manifested the disease in 2002 and 2010, respectively. Notably, five of the six patients with anti-MDA-5 antibodies whose disease began in 2002 and all four of the patients with anti-MDA-5 antibodies whose disease began in 2010 were from these two areas (Figure 1).

**Table 2:** Population of the area of residence and the presence of CADM or anti-MDA-5 antibodies

			CADM			α-MDA-5-positive		
			
Population of area of residence (×1,000)	Total number of DM patients (M:F)	Mean age at onset (range)	Number (%) of patients (M:F)	*P *value*	Mean age at onset (range)	Number (%) of patients (M:F)	*P *value**	Mean age at onset (range)
T1 (0.5 to 108)	31 (4:27)	49.0 (4 to 70)	16 (51.6%) (1:15)	*P *for difference = 0.096	47.3 (4 to 69)	14 (45.2%) (1:13)	*P *for difference = 0.012	48.8 (20 to 66)
T2 (130 to 826)	26 (7:19)	44.0 (9 to 80)	10 (38.5%) (3:7)	*P *for trend = 0.031	40.0 (9 to 59)	7 (26.9%) (3:4)	*P *for trend = 0.003	39.9 (11 to 59)
T3 (2,200)	38 (17:21)	47.3 (1 to 79)	10 (26.3%) (4:6)		45.9 (1 to 73)	5 (13.2%) (1:4)		51.0 (39 to 63)

CADM, clinically amyopathic dermatomyositis; DM, dermatomyositis; M:F, male:female; MDA-5, melanoma differentiation-associated gene 5. *Prevalence of CADM in total DM. **Prevalence of anti-MDA-5 in total DM.

### Seasonal onset

The information on seasonality of disease onset was available for 78 patients, including 33 CADM and 25 anti-MDA-5-positive patients. There were no significant seasonal patterns of disease onset in the overall patient group or in the subgroups of male, female, CADM or anti-MDA-5-positive patients (data not shown). However, the incidence of anti-MDA-5 antibodies in areas with populations under 108 × 10^3^, but not in areas with populations over 130 × 10^3^, was the highest in autumn (onset in autumn in areas with populations under 108 × 10^3 ^vs. onset in autumn in areas with populations over 130 × 10^3 ^= 8/14 vs. 1/11, *P *= 0.033).

## Discussion

A Japanese multicenter study confirmed recently that patients with anti-MDA-5 antibodies frequently have CADM with rapidly progressive ILD and a poor prognosis [15]. With increasing awareness of the CADM disease subtype, which was proposed by Sontheimer in the 1990s, we felt not only that the prevalence of CADM is increasing but also that more CADM patients with anti-MDA-5 antibodies have recently been coming from rural areas than from urban ones. To examine these matters statistically, we investigated the prevalence of CADM and anti-MDA-5 antibodies among all of the DM patients.

Because the present study was neither population based nor community based, it is difficult to say that the incidence of CADM is increasing. However, the frequency of anti-MDA-5 antibodies among all DM patients is increasing. Although this autoantibody was only recently characterized [8,9], our initial study found that the serum collected from one patient in 1994 was anti-MDA-5 antibody-positive. Contrary to the increasing prevalence of anti-MDA-5 antibodies, other types of autoantibodies appear to be decreasing. We also characterized the prevalence of anti-transcriptional intermediary factor-1γ antibodies among all patients examined in this study. These antibodies, however, which were detected in 12 patients, showed no significant epidemiological characteristics under the same analysis (data not shown). In our previous study using traditional immunoprecipitation experiments, we did not detect significant decreases in the prevalence of any specific autoantibodies [11]. There is little possibility that the long storage of the sera caused the autoantibodies to become less active, however, because various kinds of DM/polymyositis-specific autoantibodies were found in many of the sera that were drawn in 1994 and 1995 (two patients with anti-transcriptional intermediary factor 1γ, two patients with anti-MJ, two patients with anti-PL-7, one patient with anti-Jo-1, one patient with anti-EJ and one patient with anti-KS; our unpublished observations), along with the anti-MDA-5 antibodies found in the two other patients during this period.

MDA-5 detects some viruses, including picornaviruses, and is involved in the host defense response to infection. Antibodies to coxsackievirus B, a picornavirus, were previously reported to be prevalent in patients with juvenile DM [16]. Although we could not find an epidemiologic study on the environmental levels of picornavirus in our district, the seasonal distribution of viruses in the river water in Nara Prefecture, which is also in central Japan, has been examined [17]. The coxsackievirus B levels peaked there in the summer, and the virus continued to be detected in the autumn and winter. Interestingly, there was a marked increase in the prevalence of anti-MDA-5 antibodies in our study in areas northeast and northwest of Nagoya (Figure 2). These regions are on the Kiso River, which is the biggest river in our area (blue line in Figure 2). In these areas, there was also an accumulation of CADM. It is unlikely that sun exposure strongly contributed to the pathogenesis, because the 15 patients with CADM included only one outdoor worker.

The present study has several limitations because of the small number of study subjects. The time lag between the initial presentation of disease and the clinical assessment should be considered. The interval between disease onset and the time of sera collection in this study was not significantly different, however, between patients with and without CADM, between patients with and without anti-MDA-5 antibodies, or among the tertiles depicted in Table 1 (data not shown), suggesting that the patient follow-up periods did not differ by disease subtype. Since people in rural areas generally have reduced access to specialists, patients with severe illness, such as anti-MDA-5-positive patients, might be more prevalent in rural areas than in urban areas. Moreover, medical practices at a university hospital have an inherent referral bias.

Many reports have suggested that environmental factors play a role in the development of DM and the production of myositis-related autoantibodies (reviewed in [18]). No single factor, however, can explain that development and that production, and the possible growing prevalence of CADM and anti-MDA-5-positive patients. It seems difficult to identify environmental factors that possibly increase the annual prevalence of CADM and anti-MDA-5-positive patients, because patients could have several environmental exposures that have possible interrelationships with genetic risk factors. Various environmental exposures require confirmation in case-controlled studies to determine which are associated with disease onset and whether these play any role in etiology.

To our knowledge, this is the first epidemiologic study on anti-MDA-5 antibodies. Although it is difficult to draw strong conclusion from a single cohort study, epidemiologic studies play an important role in disease assessment. These studies determine the extent of disease and the natural history within a community, identify potential etiologic factors and enhance our understanding of disease pathogenesis.

## Conclusions

Clinically amyopathic dermatomyositis might be growing in prevalence with the increase of anti-MDA-5 antibody-positive patients in central Japan. Regional differences in the incidences of the anti-MDA-5 antibody would suggest that environmental factors contribute to the production of autoantibodies against MDA-5. It will be important to conduct larger population-based studies through multicenter collaboration using DM-specific autoantibodies to define patient groups and clarify the disease etiology associated with environmental factors.

## Abbreviations

CADM: clinically amyopathic dermatomyositis; DM: dermatomyositis; ILD: interstitial lung disease; MDA-5: melanoma differentiation-associated gene 5.

## Competing interests

The authors declare that they have no competing interests.

## Authors' contributions

YM, KS and KH organized the patient registry. YM and KH performed laboratory assays. KT participated in the design of the study and performed the statistical analysis. YM conceived of the study design and wrote the manuscript with input and consensus from all authors. KS and MA participated in the coordination of the study and helped to draft the manuscript. All authors read and approved the final manuscript.

## References

[B1] Leff RL Burgess SH Miller FW Love LA Targoff IN Dalakas MC Joffe MM Plotz PH Distinct seasonal patterns in the onset of adult idiopathic inflammatory myopathy in patients with anti-Jo-1 and anti-signal recognition particle autoantibodies Arthritis Rheum 1991 34 1391 1396 1953817 10.1002/art.1780341108

[B2] Sarkar K Weinberg CR Oddis CV Medsger TA Jr Plotz PH Reveille JD Arnett FC Targoff IN Genth E Love LA Miller FW Seasonal influence on the onset of idiopathic inflammatory myopathies in serologically defined groups Arthritis Rheum 2005 52 2433 2438 10.1002/art.21198 16052581

[B3] Phillips BA Zilko PJ Garlepp MJ Mastaglia FL Seasonal occurrence of relapses in inflammatory myopathies: a preliminary study J Neurol 2002 249 441 444 10.1007/s004150200036 11967650

[B4] Okada S Weatherhead E Targoff IN Wesley R Miller FW International Myositis Collaborative Study Group Global surface ultraviolet radiation intensity may modulate the clinical and immunologic expression of autoimmune muscle disease Arthritis Rheum 2003 48 2285 2293 10.1002/art.11090 12905483

[B5] Love LA Weinberg CR McConnaughey DR Oddis CV Medsger TA Jr Reveille JD Arnett FC Targoff IN Miller FW Ultraviolet radiation intensity predicts the relative distribution of dermatomyositis and anti-Mi-2 autoantibodies in women Arthritis Rheum 2009 60 2499 2504 10.1002/art.24702 19644877 PMC2855681

[B6] Sontheimer RD Would a new name hasten the acceptance of amyopathic dermatomyositis (dermatomyositis siné myositis) as a distinctive subset within the idiopathic inflammatory dermatomyopathies spectrum of clinical illness? J Am Acad Dermatol 2002 46 626 636 10.1067/mjd.2002.120621 11907524

[B7] Sato S Kuwana M Clinically amyopathic dermatomyositis Curr Opin Rheumatol 2010 22 639 643 10.1097/BOR.0b013e32833f1987 20827200

[B8] Sato S Hirakata M Kuwana M Suwa A Inada S Mimori T Nishikawa T Oddis CV Ikeda Y Autoantibodies to a 140-kd polypeptide, CADM-140, in Japanese patients with clinically amyopathic dermatomyositis Arthritis Rheum 2005 52 1571 1576 10.1002/art.21023 15880816

[B9] Sato S Hoshino K Satoh T Fujita T Kawakami Y Fujita T Kuwana M RNA helicase encoded by melanoma differentiation-associated gene 5 is a major autoantigen in patients with clinically amyopathic dermatomyositis: association with rapidly progressive interstitial lung disease Arthritis Rheum 2009 60 2193 2200 10.1002/art.24621 19565506

[B10] Nakashima R Imura Y Kobayashi S Yukawa N Yoshifuji H Nojima T Kawabata D Ohmura K Usui T Fujii T Okawa K Mimori T The RIG-I-like receptor IFIH1/MDA5 is a dermatomyositis-specific autoantigen identified by the anti-CADM-140 antibody Rheumatology (Oxford) 2010 49 433 440 10.1093/rheumatology/kep375 20015976

[B11] Hoshino K Muro Y Sugiura K Tomita Y Nakashima R Mimori T Anti-MDA5 and anti-TIF1-γ antibodies have clinical significance for patients with dermatomyositis Rheumatology (Oxford) 2010 49 1726 1733 10.1093/rheumatology/keq153 20501546

[B12] Kato H Takeuchi O Sato S Yoneyama M Yamamoto M Matsui K Uematsu S Jung A Kawai T Ishii KJ Yamaguchi O Otsu K Tsujimura T Koh CS Reis e Sousa C Matsuura Y Fujita T Akira S Differential roles of MDA5 and RIG-I helicases in the recognition of RNA viruses Nature 2006 441 101 105 10.1038/nature04734 16625202

[B13] Bohan A Peter JB Bowman RL Pearson CM A computer-assisted analysis of 153 patients with polymyositis and dermatomyositis Medicine (Baltimore) 1977 56 255 286 327194 10.1097/00005792-197707000-00001

[B14] Kobayashi I Okura Y Yamada M Kawamura N Kuwana M Ariga T Anti-melanoma differentiation-associated gene 5 antibody is a diagnostic and predictive marker for interstitial lung diseases associated with juvenile dermatomyositis J Pediatr 2011 158 675 677 10.1016/j.jpeds.2010.11.033 21232756

[B15] Hamaguchi Y Kuwana M Hoshino K Hasegawa M Kaji K Matsushita T Komura K Nakamura M Kodera M Suga N Higashi A Ogusu K Tsutsui K Furusaki A Tanabe H Sasaoka S Muro Y Yoshikawa M Ishiguro N Ayano M Muroi E Fujikawa K Umeda Y Kawase M Mabuchi E Asano Y Sodemoto K Seishima M Yamada H Sato S Takehara K Fujimoto M Clinical correlations with dermatomyositis-specific autoantibodies in adult Japanese patients with dermatomyositis: a multicenter cross-sectional study Arch Dermatol 2011 147 391 398 10.1001/archdermatol.2011.52 21482889

[B16] Christensen ML Pachman LM Schneiderman R Patel DC Friedman JM Prevalence of Coxsackie B virus antibodies in patients with juvenile dermatomyositis Arthritis Rheum 1986 29 1365 1370 10.1002/art.1780291109 3022759

[B17] Tani N Dohi Y Kurumatani N Yonemasu K Seasonal distribution of adenoviruses, enteroviruses and reoviruses in urban river water Microbiol Immunol 1995 39 577 580 7494496 10.1111/j.1348-0421.1995.tb02245.x

[B18] Prieto S Grau JM The geoepidemiology of autoimmune muscle disease Autoimmun Rev 2010 9 A330 A334 10.1016/j.autrev.2009.11.006 19906360

